# Trends in (LaMnO_3_)_*n*_/(SrTiO_3_)_*m*_ superlattices with varying layer thicknesses

**DOI:** 10.1038/srep13762

**Published:** 2015-09-01

**Authors:** J. Jilili, F. Cossu, U. Schwingenschlögl

**Affiliations:** 1 KAUST, PSE Division, Thuwal 23955-6900, Kingdom of Saudi Arabia

## Abstract

We investigate the thickness dependence of the structural, electronic, and magnetic properties of (LaMnO_3_)_*n*_/(SrTiO_3_)_*m*_ (*n*, *m* = 2, 4, 6, 8) superlattices using density functional theory. The electronic structure turns out to be highly sensitive to the onsite Coulomb interaction. In contrast to bulk SrTiO_3_, strongly distorted O octahedra are observed in the SrTiO_3_ layers with a systematic off centering of the Ti atoms. The systems favour ferromagnetic spin ordering rather than the antiferromagnetic spin ordering of bulk LaMnO_3_ and all show half-metallicity, while a systematic reduction of the minority spin band gaps as a function of the LaMnO_3_ and SrTiO_3_ layer thicknesses originates from modifications of the Ti *d*_*xy*_ states.

A zoo of extraordinary phenomena, such as two dimensional quantum gases^1^, superconductivity^2^, magnetism^3^, magnetoresistance^4^, exchange bias^5^,^6^, half-metallicity^7^, and ferroelectricity^8^, is hosted by perovskite oxide heterostructures. The lattice, charge, orbital, and spin degrees of freedom and their competition give rise to these properties^9^. In general, electrostatic and strain effects drive structural and electronic reconstructions at interfaces^10^, which thus often behave distinctly different from their component materials. Nowadays the thickness of superlattices can be determined and controlled down to the atomic scale, where interface effects become more and more important. Thus, the thickness is a key factor for tailoring the electronic and transport properties. For example, Nanda and coworkers^11^ have studied the magnetism in LaMnO_3_/SrMnO_3_ superlattices with varying layer thicknesses, concluding that the charge reconstruction is confined to two unit cells around the interfaces. Volume changes have been observed in NaNbO_3_/SrTiO_3_ superlattices^12^, for example, and it has been demonstrated that the appearance of metallicity correlates with the layer thicknesses^13^.

Manganites and titanates are popular perovskite oxides, displaying colossal magnetoresistance^14^ and superconductivity^15^. Colossal magnetoresistance was first observed in 1994 in La_1−*x*_Ca_*x*_MnO_3_^16^ and gained considerable attention thereafter. The parent compound, LaMnO_3_, is an antiferromagnetic insulator, which becomes ferromagnetic under hole doping with Ca^17^, Sr^18^, and Ba^19^. Therefore, superlattices with SrTiO_3_ can be expected to show an unusual magnetic behavior and accordingly are attracting interest both for fundamental and technological reasons. The authors of Ref. 20, for example, have reported significant doping effects in LaMnO_3_/SrTiO_3_ superlattices, which can extend over few unit cells^21^, and have studied their origin. In LaSrMnO_3_/SrTiO_3_ superlattices with SrTiO_3_ layers thinner than 1 nm an interfacial charge transfer leads to finite Ti magnetic moments^22^. In general, the thicknesses of the component layers in a LaMnO_3_/SrTiO_3_ superlattice seem to strongly influence the magnetic and transport properties^23^. This would be interesting, because the behavior of the system could be controlled by means of the layer thickness. However, it requires detailed understanding of the strong lattice distortions and their implications for the physics of this superlattice, which is the topic of the present study.

## Results

**Figure 1:**
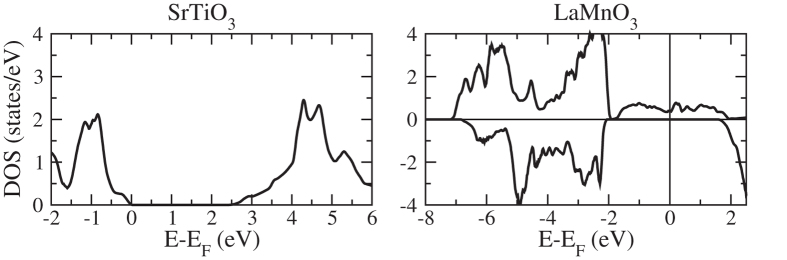
Total densities of states of bulk SrTiO_3_ and strained bulk LaMnO_3_ .

(LaMnO_3_)_*n*_/(SrTiO_3_)_*m*_ superlattices are modeled by a 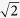  ×  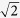 in-plane supercell of the cubic perovskite structure for taking into account possible effects of octahedral rotations. We use the Vienna Ab-initio Simulation Package^24^ with projector augmented wave pseudopotentials and employ the generalized gradient approximation in the parametrization of Perdew, Burke, and Ernzerhof (including spin polarization). The density of states is calculated by the tetrahedron method (smearing 0.01 eV) with Blöchl corrections^25^. Because of the correlated nature of the transition metal *d* orbitals, we add an onsite Coulomb interaction using the Lichtenstein scheme^26^. The values for the *U* and *J* parameters are taken from Refs 27, 28, 29 as 4 eV and 1 eV for the Mn *d* states, 5 eV and 0.5 eV for the Ti *d* states, and 9 eV and 1 eV for the La *f* states. We have tested for the bulk compounds that these values are transferable to the parametrization of Perdew, Burke, and Ernzerhof by comparing the densities of states, which show no qualitative difference. The optimized lattice constant of SrTiO_3_ is 3.97 Å, with a band gap of 2.6 eV, and the optimized in-plane and out-of-plane lattice constants of LaMnO_3_ are 3.95 Å and 4.01 Å. The total densities of states obtained for bulk SrTiO_3_ and strained bulk LaMnO_3_ (half-metallic) are shown in Fig. 1.

**Figure 2:**
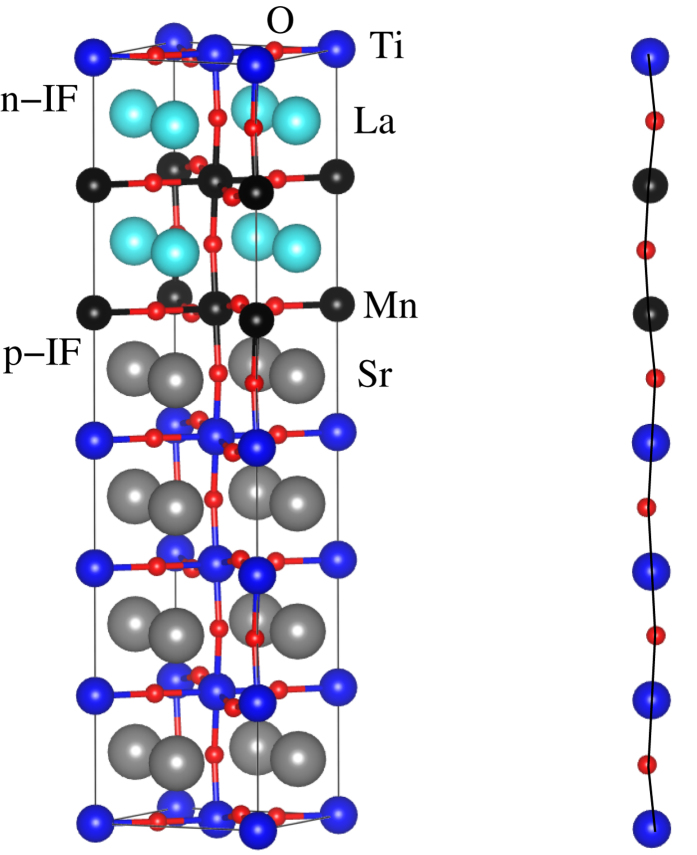
Left: Structure of the (LaMnO_3_)_*n*_/(SrTiO_3_)_*m*_ superlattice for *n*:*m* = 2:4. Right: Schematic view of the Mn-O and Ti-O bonds perpendicular to the interfaces.

Structural optimization of the superlattices with and without onsite interaction is found to yield substantial differences in the electronic structure, especially near the Fermi level. More specifically, the minority channel shows a metallic character without onsite interaction for *n*:*m* = 2:2 (2 unit cells of LaMnO_3_ alternate with 2 unit cells of SrTiO_3_), whereas with onsite interaction we obtain a half-metallic character. The supercells have C_2*h*_ point group symmetry (tetragonal perovskite structure). An optimized in-plane lattice constant of 5.56 Å is obtained for the 2:2 system and is used for the larger superlattices. The out-of-plane lattice constant is optimized individually in each case. Starting from the 2:2 system, we fix the LaMnO_3_ thickness and increase the SrTiO_3_ thickness (2:4, 2:6, 2:8) or fix the SrTiO_3_ thickness and increase the LaMnO_3_ thickness (4:2, 6:2, 8:2). The 4:4 system is also considered for comparison. Figure 2(left) illustrates the 2:4 system as an example. There are two types of interfaces, the *n*-type LaO/TiO_2_ interface and the *p*-type SrO/MnO^2^ interface, denoted in the following as n-IF and p-IF, respectively. The nomenclature refers to the compensating charges formed at the 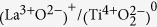 and 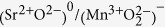 contacts, respectively.

The Ti-O-Mn bond angle at the n-IF (where the MnO_6_ and TiO_6_ octahedra are connected) is found to be 159.4°, whereas at the p-IF it has a larger value of 164.8°, which corresponds to a reduced tilting. While substantial modifications of the structure are expected in the vicinity of an interface, strong effects are also observed for the O octahedra further apart. For instance, the Mn-O-Mn bond angles increase by up to 8.3° with respect to the bulk value (154.6°), which supports double exchange. On the other hand, the Ti-O-Ti bond angles strongly decrease throughout the SrTiO_3_ layer, reflecting huge distortions as compared to the bulk cubic structure. In general, the Ti-O-Ti (Mn-O-Mn) bond angles near the p-IF (n-IF) are less modified, because the distance to the interface is larger. The tiltings of the O octahedra are found to vary distinctly for different thicknesses *n*:*m*, and so do the electronic properties.

For increasing *m* the Mn-O (Ti-O) bond length along the *z* axis at the p-IF decreases (increases) from 2.13 Å to 1.99 Å (1.91 Å to 1.97 Å), while at the n-IF the Mn-O bond length stays close to 2.13 Å and the Ti-O bond length decreases from 2.04 Å to 1.95 Å, see Table 1. A schematic view of the Mn-O and Ti-O bonds is given in Fig. 2(right). For increasing *n* the Mn-O bond length at the p-IF maintains a value of 2.10 Å, while the Ti-O bond length decreases (bulk value 1.95 Å) from 1.91 Å in the 2:2 system to 1.87 Å in the 8:2 system. At the n-IF the Mn-O bond length shows no clear trend, while the Ti-O bond length grows significantly from 2.04 Å in the 2:2 system to 2.08 Å in the 8:2 system. It is found that all Ti atoms shift systematically off the center of their O octahedron towards the p-IF by up to 0.1 Å (in all cases more at the p-IF than at the n-IF), implying that a permanent dipole is created, while bulk SrTiO_3_ is not ferroelectric. The off-centering is generally enhanced at the interfaces when *n* increases and reduced when *m* increases. It is known that the properties of SrTiO_3_ are very sensitive to dopants and external perturbations^30^ so that it cannot surprise that the superlattices react similarly^31^. We also note that we obtain for bulk SrTiO_3_ a ferroelectric distortion of 0.03 Å, which is a known artefact of the employed methodology^32^. However, this effect is significantly smaller than the off-centerings described above and therefore does not affect our conclusions.

**Figure 3:**
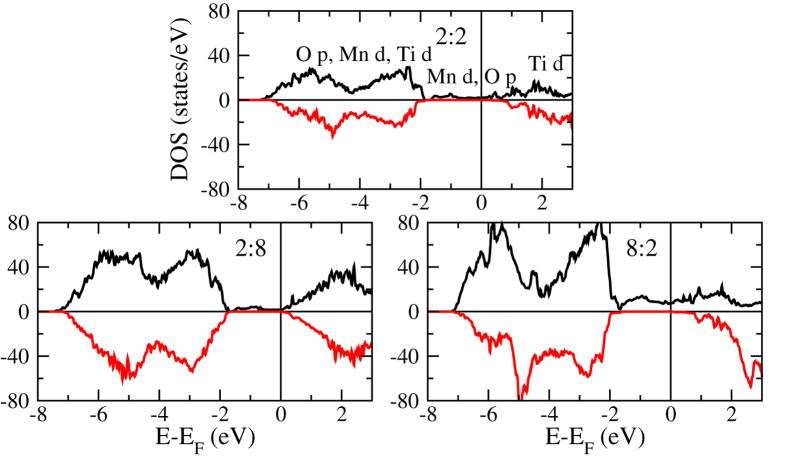
Total densities of states for thicknesses n:m = 2:2, 2:8, and 8:2.

**Figure 4:**
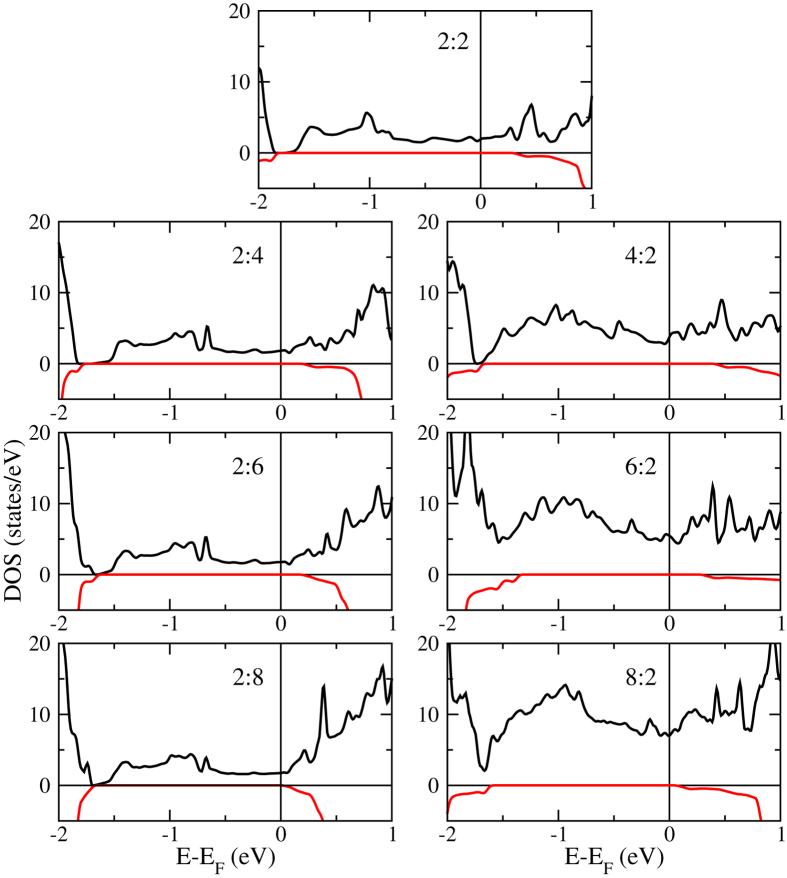
Total densities of states near the Fermi energy for thicknesses *n*:*m* = 2:2, 2:4, 2:6, 2:8, 4:2, 6:2, and 8:2.

Bulk LaMnO_3_ is an A-type antiferromagnetic (AFM) Mott insulator, due to a combination of superexchange and Zener double exchange^33^, whereas SrTiO_3_ is a non-magnetic insulator (d^0^ configuration of Ti). Figure 3 shows the total densities of states obtained for the 2:2, 2:8, and 8:2 systems. The general shape of the curves is similar, showing half-metallicity in each case. From −7 to −2 eV we find mainly Mn *d*, Ti *d*, and O *p* states, from −2 to 2 eV Mn *d* and O *p* states, and above 2 eV Ti *d*, La *f*, Sr *d*, and O *p* states. Figure 4 demonstrates systematic changes for varying *n* and *m*. The density of states at the Fermi energy remains similar for growing *m* but increases significantly for growing *n* due to the fact that these states mainly belong to the LaMnO_3_ layer. Importantly, for increasing *n* as well as *m*, the minority spin band gap is reduced substantially. Indeed, the band onsets in the 2:8 and 8:2 systems are close to the Fermi energy so that the half-metallicity will most likely vanish for *n*, *m* > 8.

**Figure 5:**
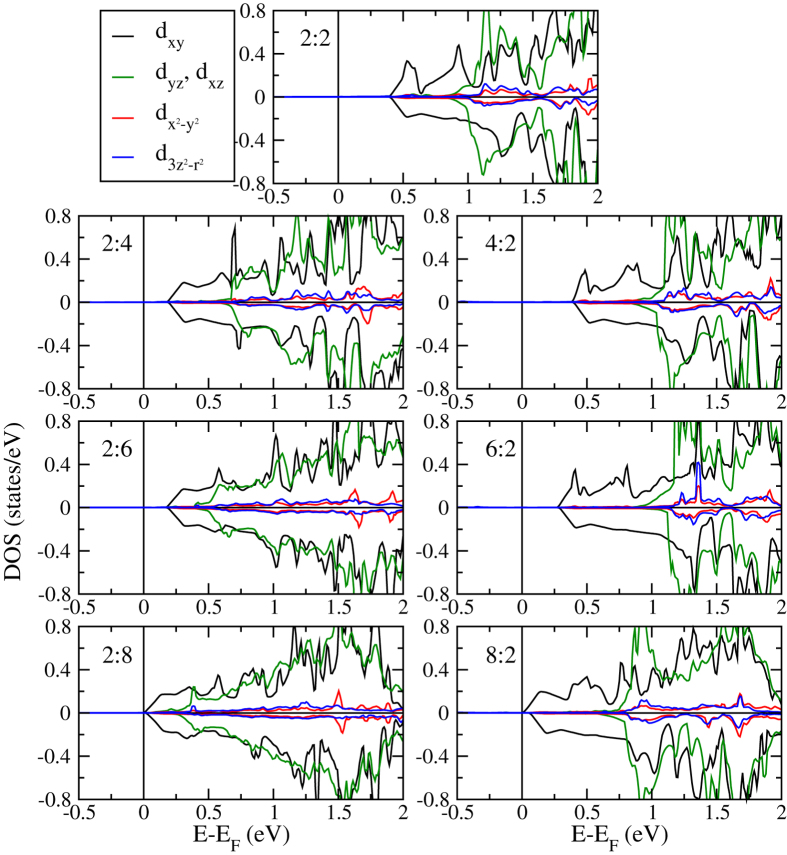
Projected densities of states of the Ti atom next to the n-IF for thicknesses *n*:*m* = 2:2, 2:4, 2:6, 2:8, 4:2, 6:2, and 8:2.

**Figure 6:**
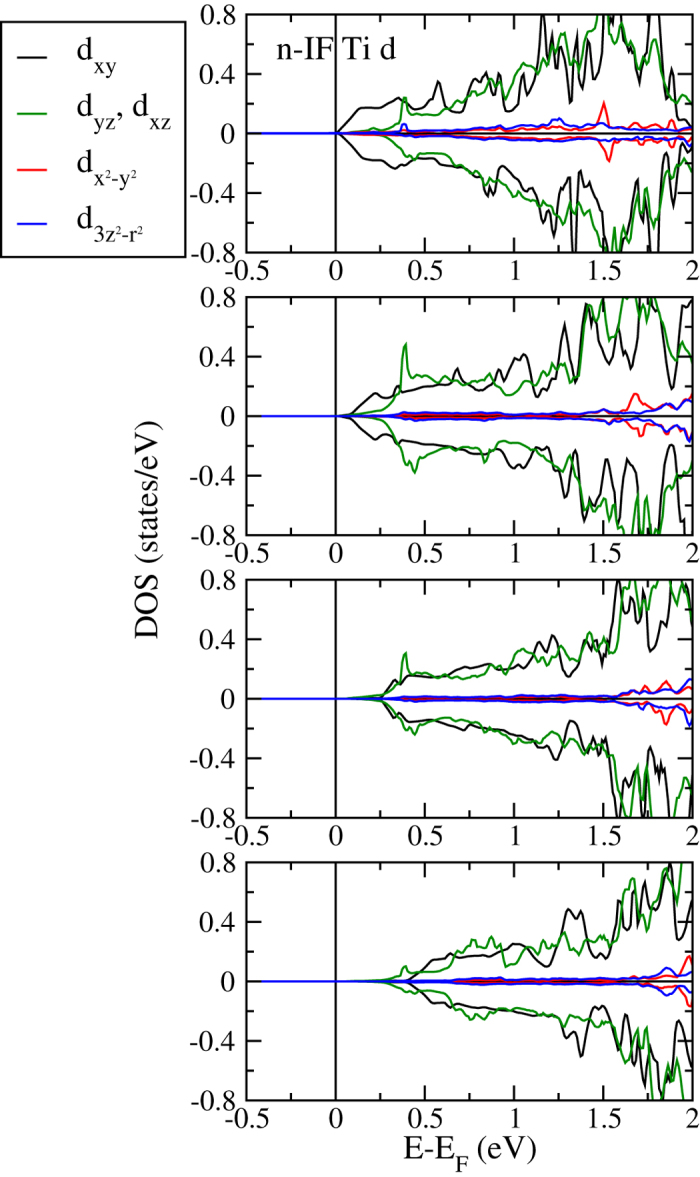
Projected densities of states of the Ti atoms in layers with increasing distance from the 2:8 n-IF.

**Figure 7:**
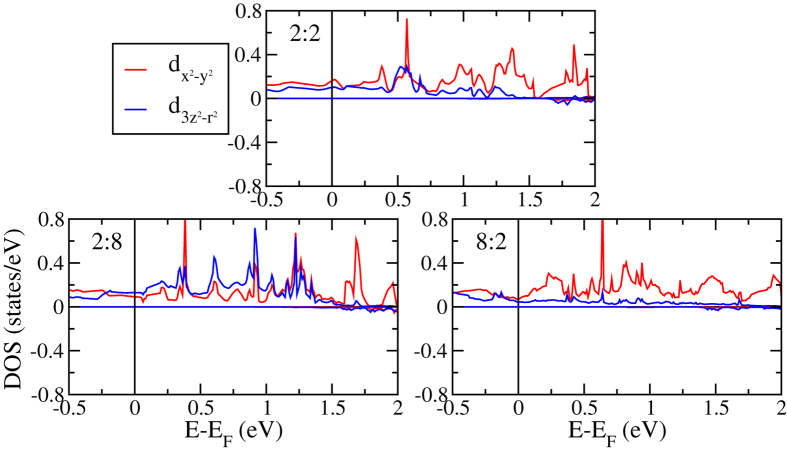
Projected densities of states of the Mn atom next to the p-IF for thicknesses *n*:*m* = 2:2, 2:8, and 8:2.

**Figure 8:**
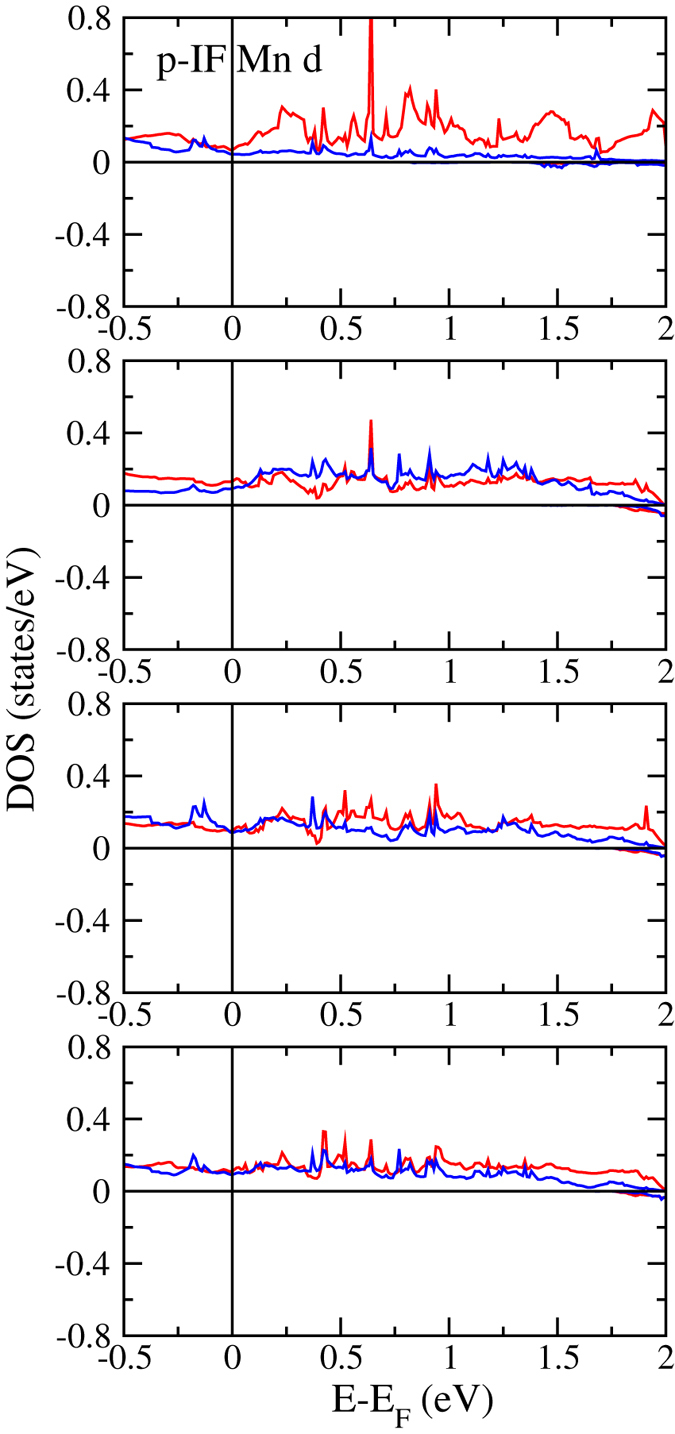
Projected densities of states of the Mn atoms in layers with increasing distance from the 8:2 p-IF.

The reduction of the minority spin band gap is explained by the projected densities of states of the Ti atoms at the n-IF in Fig. 5. The Ti 3*d* orbitals, being split into *d*_*xy*_, *d*_*yz*_, *d*_*xz*_ (*t*_2*g*_) and 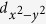, 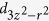 (*e*_*g*_) states due to the octahedral crystal field, systematically shift to lower energy for increasing *n* and *m*, which is consistent with previous experimental observations for LaAlO_3_/SrTiO_3_ interfaces^34^. The energetic lowering is strongest for the *d*_*xy*_ states, which thus govern the reduction of the minority spin band gap. The orbital ordering seen in Fig. 5 decreases for increasing *m*, while it remains similar for increasing *n*. In addition, it is always more pronounced at the n-IF than at the p-IF, and almost lost at the p-IF and in the bulk-like regions of the 2:8 system. Figure 6 gives projected densities of states of Ti atoms in layers with increasing distance from the 2:8 n-IF. As to be expected, away from the n-IF the states shift to higher energy. Projected densities of states of the Mn atoms at the p-IF are shown in Fig. 7. Note that the Mn 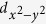 and 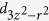 orbitals contribute at the Fermi energy in the majority spin channel, though differently for the different systems. Since bulk LaMnO_3_ forced into C_2*h*_ symmetry becomes metallic, the conducting Mn states are a result of the in-plane strain. The metallicity at the p-IF is substantially reduced for increasing *n*, especially in the 8:2 system, as the LaMnO_3_ layer becomes more bulk-like. Figure 8 gives projected densities of states of the Mn atoms in layers with increasing distance from the 8:2 p-IF. The distinct difference between the 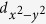 and 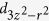 states is reduced when the distance increases.

In order to assess the magnetic ground state, we study the total energies for FM and A-type AFM spin ordering for strained bulk LaMnO_3_ and the superlattices. Other spin orderings (such as C-type and G-type AFM) have much higher energies, as we have checked for the 2:2 system. FM spin ordering is found to be favorable, in agreement with previous experiments^20^,^23^,^34^ and calculations^35^ as well as reports on related perovskite oxide interfaces^36^,^37^,^38^. The energy difference (per formula unit) between FM and A-type AFM spin ordering is 0.75 eV for strained bulk LaMnO_3_, whereas in the case of the superlattices it slightly decreases from 0.27 eV to 0.20 eV for increasing *m* and remarkably increases from 0.27 eV to 0.85 eV for increasing *n*, which shows that it can be attributed to the LaMnO^3^ layer (the Ti magnetic moments are very small). Because of this, we normalize our results in the following with respect to the number of Mn atoms. The energy difference steadily decreases for increasing *n* as well as *m* though the FM ordering, which is due to Zener double exchange between partially filled Mn *e*_*g*_ states^39^, remains favorable. For *x* = 0.3 FM spin ordering is also established in La_1−*x*_Ca_*x*_MnO_3_, see the neutron diffraction experiments in Ref. 40. Moreover, X-ray magnetic circular dichroism and absorption spectroscopy on LaAlO_3_/SrTiO_3_ have given direct evidence of a FM interface state, originating from the Ti *d*_*xy*_ orbitals^34^. Interestingly, induced Ti magnetic moments of 0.02 to 0.04 *μ*_*B*_ are observed at both interfaces in our systems, see Table 2, whereas far from the interfaces they are negligible (<0.01 *μ*_*B*_). For increasing *m* they increase (decrease) at the n-IF (p-IF), while for increasing *n* we obtain higher magnetic moments at the p-IF (~0.04 *μ*_*B*_) than at the n-IF (~0.02 *μ*_*B*_). We note that even small Ti magnetic moments at perovskite oxide interfaces can have important consequences for the magnetism^34^,^41^.

We find Mn magnetic moments of 3.72 to 4.03 *μ*_*B*_, with larger values at the n-IF than at the p-IF in all cases. When *m* increases they increase (decrease) at the n-IF (p-IF), while the changes are not systematic under variation of *n*. The highest value appears in the 4:4 system at the n-IF. Concerning the charges in the atomic orbitals, we find small but finite differences from the corresponding bulk values at both interfaces, see Table 2. A detailed analysis shows that for increasing *n* (*m*) the Mn atoms lose more (less) charge at the p-IF. Moreover, the Ti (Mn) atoms gain (lose) charge at the n-IF (p-IF) in all cases, while there is no clear trend at the p-IF (n-IF) because of the larger distances of the atoms from to the interface plane. The observed small charge transfers give rise to the mentioned Ti magnetic moments but cannot explain the ferroelectric distortions in the SrTiO_3_ layer, which thus are likely direct consequences of the interface interaction.

## Discussion

The structural, electronic, and magnetic properties of (LaMnO_3_)_*n*_/(SrTiO_3_)_*m*_ superlattices have been investigated and compared for various thicknesses *n*:*m*. Both *n* and *m* are found to strongly affect the material properties. In general, the SrTiO_3_ layers show heavy distortions of the O octahedra, which are not present in bulk SrTiO_3_, consistent with recent experimental findings^42^. Since LaMnO_3_ is polar along the [001] direction (with alternating (LaO)^+^ and (MnO_2_)^−^ layers) and SrTiO_3_ is non-polar, a compensation mechanism is required. As the strained LaMnO_3_ is metallic, we could expect accumulation of charge carriers at the interfaces. However, this effect is much too small according to the charge deviations reported in Table 2 so that a second mechanism must play a role. The projected densities of states of the Ti atoms in layers of increasing distance from the 2:8 n-IF in Fig. 6 show an almost continuous shift of all curves to higher energy. This reflects the built up of an electric field, which in turn induces ferroelectric distortions in the SrTiO_3_ region, as discussed before. The ferroelectric distortions constitute the main compensation mechanism in the present superlattice. Similar distortions of the TiO_6_ octahedra have been reported in Ref. 28.

All studied systems exhibit half-metallicity, though our results indicate that this property will vanish for *n*, *m* > 8. FM spin ordering is always energetically favorable over AFM spin ordering but the preference becomes smaller for increasing *n*, since the A-type AFM ordering of bulk LaMnO_3_ starts to dominate. The Ti magnetic moments observed at the interfaces are small. The authors of Ref. 43 have found ferromagnetic and antiferromagnetic alignment of the Mn and Ti spins for *n*:*m* ratios of 3:2 and 17:2, respectively, and have argued that Mn-O-Ti superexchange therefore must play a role. In general, defects and cation intermixing are important for the stabilization of perovskite superlattices. For instance, the high electrical conductivity of the LaAlO_3_/SrTiO_3_ interface is related to the formation of O vacancies during the deposition process due to the extra charges introduced into the system^44^. In addition, Sr/La intermixing leads to a metallic interface between the insulators LaTiO_3_ and SrTiO_3_, because mixed valent Ti states are created^45^. We expect that these factors are less important for the LaMnO_3_/SrTiO_3_ interface, as it was found to be rather sharp^42^. We find a systematic reduction of the minority spin band gaps with increasing *n* and *m*, which originates mainly from an energetic downshift of the Ti *d*_*xy*_ states.

## Methods

The atomic sphere radii are chosen such that overlap is avoided during the ionic relaxation, for which we set the energy tolerance to 10^−3^ eV, employ a 4 × 4 × 1 k-mesh, a Gaussian smearing of 0.05 eV, and an energy cutoff of 500 eV. To obtain accurate electronic states, we set the energy tolerance to 10^−5^ eV and adopt dense k-meshes of 8 × 8 × 3 for *n* + *m* = 4, 8 × 8 × 2 for *n* + *m* = 6, and 8 × 8 × 1 for *n* + *m* = 8 and 10.

## Additional Information

**How to cite this article**: Jilili, J. *et al.* Trends in (LaMnO_3_)*n*/(SrTiO_3_)*m* superlattices with varying layer thicknesses. *Sci. Rep.* **5**, 13762; doi: 10.1038/srep13762 (2015).

## Figures and Tables

**Table 1:** Bond distances (in Å) at the n-IF and p-IF for different *n*:*m*.

		***n*-IF**	***p*-IF**		***n*-IF**	***p*-IF**
Mn-O	2:2	2.12	2.13	4:4	2.17	2.00
Ti-O		2.04	1.91		2.01	1.90
Mn-O	2:4	2.17	2.03	4:2	2.14	2.07
Ti-O		2.00	1.93		2.04	1.88
Mn-O	2:6	2.12	2.01	6:2	2.14	2.07
Ti-O		1.95	1.96		2.04	1.91
Mn-O	2:8	2.12	1.99	8:2	2.13	2.13
Ti-O		1.95	1.97		2.08	1.87

**Table 2:** Magnetic moments (in *μ*_*B*_) and charge deviations with respect to the bulk (in electrons) of the interfacial Mn and Ti atoms for different *n*:*m*.

***n***:***m***	**magnetic moment**				**charge deviation**			
	**Mn *n*-IF**	**Mn *p*-IF**	**Ti *n*-IF**	**Ti *p*-IF**	**Mn *n*-IF**	**Mn *p*-IF**	**Ti *n*-IF**	**Ti *p*-IF**
2:2	3.87	3.86	0.01	0.03	+0.01	−0.05	+0.02	+0.01
2:4	3.95	3.76	0.02	0.02	+0.03	−0.02	+0.02	+0.01
2:6	3.96	3.74	0.02	0.02	+0.03	−0.02	+0.02	+0.01
2:8	3.97	3.72	0.03	0.02	+0.04	−0.01	+0.02	−0.01
4:4	4.03	3.78	0.02	0.03	+0.03	−0.04	+0.02	0.00
4:2	3.99	3.85	0.02	0.03	0.00	−0.04	+0.01	+0.01
6:2	4.01	3.88	0.02	0.03	−0.01	−0.07	+0.06	−0.03
8:2	3.97	3.89	0.02	0.04	+0.01	−0.07	+0.04	+0.03

Positive (negative) values represent gain (loss) of charge.
